# Innate immune dysregulation in multisystem inflammatory syndrome in children (MIS-C)

**DOI:** 10.1038/s41598-023-43390-6

**Published:** 2023-09-30

**Authors:** Johana Isaza-Correa, Laura Ryan, Lynne Kelly, John Allen, Ashanty Melo, Jennifer Jones, Dean Huggard, Emer Ryan, Cilian Ó Maoldomhnaigh, Sarah Geoghehan, Patrick Gavin, Timothy Ronan Leahy, Karina Butler, Bridget Freyne, Eleanor J. Molloy

**Affiliations:** 1 Discipline of Paediatrics, Trinity College, The University of Dublin, Dublin, Ireland; 2 Trinity Translational Medicine Institute, Trinity College Dublin, Dublin, Ireland; 3 Trinity Research in Childhood Centre (TRiCC), Trinity College Dublin, Dublin, Ireland; 4 Infectious Diseases/Immunology, Children’s Health Ireland at Crumlin, Dublin, Ireland; 5 Neonatology, Children’s Health Ireland at Crumlin, Dublin, Ireland; 6 Neurodisability, Children’s Health Ireland at Tallaght, Dublin, Ireland; 7 Neonatology, The Coombe Hospital, Dublin, Ireland; 8 Discipline of Paediatrics, Trinity Centre for Health Sciences, Children’s Hospital Ireland (CHI) at Tallaght, Tallaght University Hospital, Dublin 24, Ireland

**Keywords:** Immunology, Diseases

## Abstract

MIS-C is a systemic inflammation disorder with poorly characterised immunopathological mechanisms. We compared changes in the systemic immune response in children with MIS-C (n = 12, 5–13 years) to healthy controls (n = 14, 5–15 years). Analysis was done in whole blood treated with LPS. Expression of CD11b and Toll-like receptor-4 (TLR4) in neutrophils and monocytes were analysed by flow cytometry. Serum cytokines (IL-1β, IL-2, IL-6, IL-8, IL-10, IL-Ira, TNF-α, TNF-β, IFN-Υ, VEGF, EPO and GM-CSF) and mRNA levels of inflammasome molecules (NLRP3, ASC and IL-1β) were evaluated. Subpopulations of lymphocytes (CD3^+^, CD19^+^, CD56^+^, CD4^+^, CD8^+^, TCR Vδ1^+^, TCR Vδ2^+^) were assessed at basal levels. Absolute counts of neutrophils and NLR were high in children with MIS-C while absolute counts of lymphocytes were low. Children with MIS-C had increased levels of IL-6, IL-10, TNF-β and VEGF serum cytokines at the basal level, and significantly increased TNF-β post-LPS, compared to controls. IL-1RA and EPO decreased at baseline and post-LPS in MIS-C patients compared to controls. The percentage of CD3^+^ cells, NK cells and Vδ1 was lower while B cells were higher in children with MIS-C than in controls. Dysregulated immune response in children with MIS-C was evident and may be amenable to immunomodulation.

## Introduction

Multisystem inflammatory disease in Children (MIS-C) or Paediatric Multisystem Inflammatory Syndrome Temporally Associated with Coronavirus disease 2019 (COVID-19) (PIMS-TS) is a post-infectious complication of SARS-CoV-2, first reported between April and May 2020^1,2^. According to The World Health Organization (WHO), and The Centres for Disease Prevention and Control in Europe and the US (CDC), MIS-C presents 4–6 weeks after primary infection with SARS-Cov-2 with high fever, multi-organ dysfunction and systemic inflammation^1–5^. This presentation of hyperinflammatory shock in children has similarities to Kawasaki disease (KD), toxic shock syndrome (TSS), bacterial sepsis, and macrophage-activation syndrome (MAS)^1,2,4–8^. Over half of the patients require admission to an intensive care unit, and some will require extracorporeal membrane oxygenation (ECMO)^9,10^ but overall mortality has remained at < 2%^9^. Early recognition and treatment of children and adolescents with MIS-C, and a better understating of the pathological mechanism are essential to prevent serious complications, such as coronary artery aneurysms^10–12^.

Multiple alterations in inflammatory markers have been described in MIS-C with high neutrophil count, high neutrophil/lymphocyte ratios (NLR) and low absolute lymphocyte count similar to adults with acute COVID-19^4,7–9,13–16^. Recommended treatments include intravenous immunoglobulin (IVIG) and corticosteroids, with resistant cases responding to targeted immunomodulators, including anti-TNF and anti-IL1 agents^7,11^. Furthermore, SARS-CoV-2 proteome-wide immuno-profiling of children with MIS-C, children with COVID-19 and healthy controls revealed differential cytokine responses, IgM/IgG/IgA epitope diversity, as well as differences in antibody binding and avidity^17^. The presence of several IgG and IgA autoantigens, some of which are associated with autoimmune diseases like systemic lupus erythematosus (SLE), Sjogren's disease, polymyositis and dermatomyositis were also reported in children with MIS-C^6^. This suggests a strong immune response mediating the pathogenesis of MIS-C. This study aims to further explore the acute phase immune response in children with MIS-C/ PIMS-TS compared to healthy controls. In addition, innate immune response to endotoxin will be assessed *ex-vivo* as a measurement of ‘endotoxin tolerance’ or trained immunity, a form of innate immune memory that might indicate reduced capacity of response to an inflammatory stimulus.

## Results

### Demographics

Thirty-two children were recruited for this study, 18 clinically diagnosed with MIS-C (age range 5–13 years) and 14 healthy paediatric controls (age range 1–15 years). Clinical data was analysed from 18 children with MIS-C and all cases included in the study met the criteria for diagnosis of MIS-C as per WHO guidelines (Table 1). Four patients were COVID-19 positive at the time of diagnosis and treatment for MIS-C, and no blood samples were collected as per the biosafety restrictions of the research centre. Whole blood was collected from 26 children, including 12 children with MIS-C and 14 paediatric controls. The median age of the MIS-C cohort was 8.5 ± 3 years and 61% were males. In the healthy control group, the median age was 7.8 ± 4 years and 50% were males. The most common presenting symptoms for children with MIS-C were fever (100%), gastrointestinal affectations (89%), which usually included vomiting and/or diarrhoea and abdominal pain, a blanching polymorphous rash (72%) and cardiac symptoms/tachycardic (72%). Interestingly, 1 case (5%) did not have any known history of COVID or confirmed/suspected close contact status but otherwise met the criteria for MIS-C. In clinically collected samples, the average counts of neutrophils and lymphocytes in the MIS-C cohort were 9.43 ± 4.83 and 1.37 ± 1.07 × 10^9^ cells per litre respectively, with an average NLR of 10.05 ± 6.86 (Table S1).

**Table 1:** Demographic and clinical features of MIS-C patients.

Patients	1	2	3	4	5	6	7	8	9	10
Sex	Male	Male	Female	Male	Male	Female	Female	Female	Male	Female
Comorbidities	None	Resp Cond, GI, Complex	None	Eczema, A. Rhinitis	Asthma	Asthma	None	None	Asthma	Asthma
WHO/CDC MIS-C Criteria										
Fever	Yes	Yes	Yes	Yes	Yes	Yes	Yes	Yes	Yes	Yes
Gastrointestinal	Yes	Yes	No	Yes	Yes	Yes	Yes	Yes	Yes	Yes
Rash	Yes	Yes	No	No	Yes	No	Yes	Yes	Yes	Yes
Cardiac/Tachycardic	No	Yes	Yes	Yes	Yes	Yes	No	Yes	Yes	Yes
Hypotension/shock	No	Yes	Yes	No	Yes	Yes	No	No	Yes	No
Respiratory	No	Yes	No	No	No	No	No	Yes	No	No
Neurological	No	No	No	No	No	No	No	No	No	No
Conjunctivitis	No	No	No	No	No	No	No	No	No	No
Mucocutaneous	No	No	No	No	No	No	No	No	No	No
Cardiac Abnormalities	Normal	Normal	Abnormal	Normal	Normal	Abnormal	Abnormal	Normal	Abnormal	Normal
Troponin	–	–	103	–	–	178	30	–	63	–
BNP	–	–	–	–	–	3839	2841	–	–	–
Coagulopathy										
D-Dimer	0.75	3.65	17.5	> 10	1.78	6.92	7.19	5.74	5.25	2.94
PT	13.9	14.3	14.3	13.9	17.2	17.9	20	14.9	13.5	14.6
APTT	–	–	–	–	–	–	–	–	–	–
Inflammatory markers	Yes	Yes	Yes	Yes	Yes	Yes	Yes	Yes	Yes	Yes
ESR	55	57	–	–	–	71	–	–	–	109
CRP	52	390	122	201	293	359	108	189	265	46
PCT	–	–	–	–	–	–	–	–	–	–
Other microbial cause	No	Adeno + , Rhi/ent +	No	No	No	No	No	No	No	No
SARS-CoV-2 history										
SARS-CoV-2 PCR	Neg	Pos	Neg	Pos	Neg	Neg	Neg	Neg	Neg	Neg
Previous SARS-CoV-2 PCR	Neg	Neg	Neg	Neg	Neg	Asym Pos	Sym Pos	Neg	Sym Pos	Neg
Prior SARS-CoV-2 exposure	Suspected Contact	N/A	N/A	N/A	Contact	Contact	N/A	Suspected Contact	N/A	Suspected Contact
SARS-CoV-2 Serology	Neg	Neg	IgG +	Neg	IgG +	IgG +	Neg	Neg	IgG +	Neg
Other clinical features	Headache, malaise	No	No	Headache	Headache	No	No	Headache	Neck pain	No
Treatment	IV Ig	Steriods	Steriods	IV Ig	Steriods	Steriods	Steriods	IV Ig	Steriods	Steriods
	Aspirin	IV Ig	IV Ig	Aspirin	IV Ig	IV Ig	IV Ig	Aspirin	IV Ig	IV Ig
		Aspirin	Aspirin		Aspirin	Aspirin	Aspirin		Aspirin	Aspirin
		Remdesivir								
Admission duration (days)	9	138	6	15	12	7	6	3	8	11

Patients	11	12	13	14	15	16	17	18	Aggregate (%)	
Sex	Male	Male	Male	Male	Female	Male	Male	Female	61.1 (Male)	
Comorbidities	None	None	Asthma, Eczema	Asthma	None	None	None	Stickler	50	
WHO/CDC MIS-C Criteria										
Fever	Yes	Yes	Yes	Yes	Yes	Yes	Yes	Yes	100	
Gastrointestinal	Yes	Yes	Yes	Yes	Yes	Yes	Yes	No	88.9	
Rash	No	Yes	Yes	Yes	Yes	Yes	No	Yes	72.2	
Cardiac/Tachycardic	Yes	Yes	Yes	Yes	Yes	No	No	No	72.2	
Hypotension/shock	No	No	No	No	Yes	No	Yes	Yes	44.4	
Respiratory	No	No	No	No	No	No	No	No	11.1	
Neurological	Yes	No	No	No	No	No	Yes	No	11.1	
Conjunctivitis	No	No	No	No	No	No	No	No	0	
Mucocutaneous	No	No	No	No	No	No	No	No	0	
Cardiac Abnormalities	Abnormal	Normal	Abnormal	Abnormal	Normal	Abnormal	Abnormal	Abnormal	55.6	
Troponin	33	–	17	275	–	–	17	17		
BNP	–	–	–	2727	–	2398	4426	–		
Coagulopathy									88.9	
D-Dimer	2.67	–	1.56	–	3.8	3.7	10.43	3.61		
PT	15.1	–	14.4	–	14.2	15	15.3	13.3		
APTT	–	–	–	–	–	44	–	–		
Inflammatory markers	Yes	Yes	Yes	Yes	Yes	Yes	Yes	Yes	100	
ESR	36	86	–	49	72	140	44	24		
CRP	284	112	120	195	221	454	142	181		
PCT	–	–	–	–	1.6	–	–	–		
Other microbial cause	No	No	No	No	No	No	No	No	5.6	
SARS-CoV-2 history										
SARS-CoV-2 PCR	Pos	Pos	Neg	Neg	Neg	Neg	Neg	Neg	22.2	
Previous SARS-CoV-2 PCR	Sym Pos	Neg	Neg	Neg	Asym Pos	Sym Pos	Asym Pos	Sym Pos	44.4	
Prior SARS-CoV-2 exposure	Contact	N/A	Contact	Contact	Contact	N/A	N/A	N/A	50	
SARS-CoV-2 Serology	Neg	Neg	Neg	IgG +	Neg	Neg	IgG +	Neg	33.3	
Other clinical features	Malaise	No	No	Neck pain	Malaise	Malaise, Neck pain	Headache, neck pain	No	55.6	
Treatment	Steriods	Steriods	Steriods	Steriods	Steriods	Steriods	Steriods	Steriods	N/A	
	IV Ig	IV Ig	IV Ig	IV Ig	IV Ig	IV Ig	IV Ig	IV Ig		
	Aspirin	Aspirin	Aspirin	Aspirin	Aspirin	Aspirin	Aspirin	Aspirin		
Admission duration (days)	10	12	7	4	6	7	8	24	16 (AVER)	

*GI* gastrointestinal, *trop* troponin, *DD* D-dimer, *BNP* Brain Natriuretic Peptide, *PT* Prothrombin Time, *APTT* Activated Partial Thromboplastin Time, *ESR* Erythrocyte Sedimentation Rate, *CRP* C-reactive protein, *PCT* Procalcitonin, *Neg* Negative, *Pos* Positive, *Sym Pos* Symptomatic positive, *Asym Pos* asymptomatic Positive, *IV Ig* Intravenous Immunoglobulin Therapy, *N/A* Not Applicable. Normal reference values: Cardiac Abnormalities (Troponin < 10 ng/L; BNP < 300 pg/ml); Coagulopathy (D-Dimers < 0.5 mg/L), PT (10–12.1 s), APTT (22–41 s)); Inflammatory markers (ESR (< 20 mm/hr), CRP (< 5 mg/L), Procalcitonin (PCT; < 0.25 ng/mL)). **Reference values from RCPCH REFERENCE RANGES- 2016.

### Analysis of granulocytes and lymphocyte populations in healthy controls and children with MIS-C

**Figure 1:**
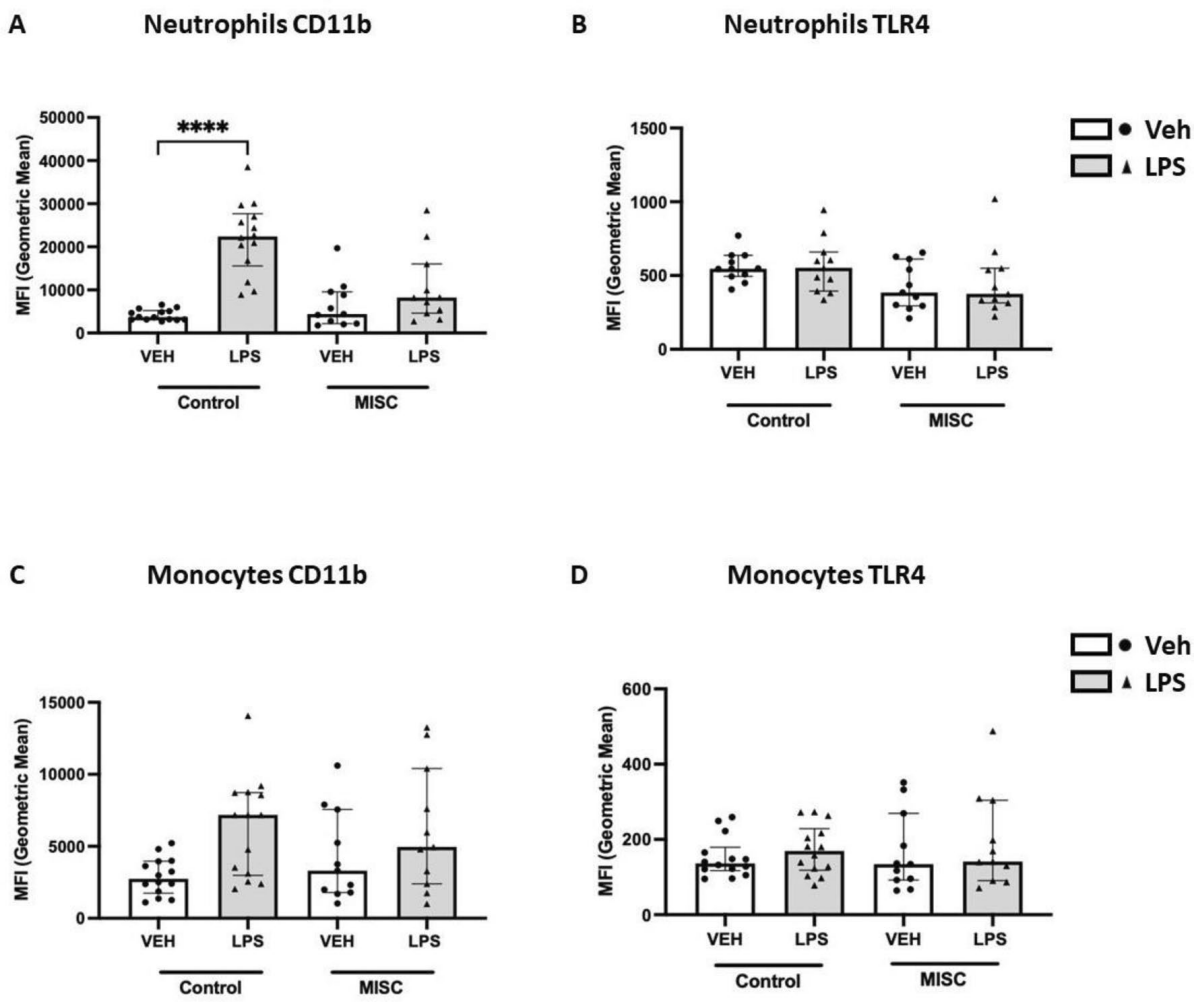
Expression of CD11b and TLR4 in neutrophils and monocytes in healthy controls and Multisystem inflammatory syndrome in children (MISC) patients: TLR4 expression in neutrophils corresponds to healthy controls (n = 11) and children with MISC (n = 11). Whole blood from both groups was stimulated with LPS (10 ng/mL) for 1 h at 37 °C, and leukocyte markers were analysed by flow cytometry. Neutrophils and monocytes were identified based on their size and granularity followed by labelling with CD66b + for granulocytes, and CD66b − and CD14 + for monocytes. (**A**, **C**) CD11b and (**B**, **D**) TLR4 expression in neutrophils and monocytes, respectively, are shown as Geometric Mean Channel Fluorescence (MFI) with median and interquartile range (IQR); “veh” refers to unstimulated (PBS). Comparisons were done between two independent cohorts and both conditions (‘Veh’ and ‘LPS-treated’). ****p < 0.0001 using Kruskal–Wallis test.

Activation of neutrophils (CD66b^+^) and monocytes (CD66b^−^ CD14^+^/CD16^+^) was evaluated by expression of cell activation and migration marker CD11b, and recognition of lipopolysaccharide (LPS) by Toll-like receptor-4 (TLR4), following LPS treatment for 1 h at 37 °C and analysed by flow cytometry. Neutrophil CD11b expression increased significantly post-LPS stimulation in controls (*p* < 0.0001) compared to the control untreated sample, but not in children with MIS-C when the post-LPS sample was compared to its corresponding untreated sample (Fig. 1A). No significant difference was observed when comparing control and children with MIS-C. Neutrophil TLR4 expression was no different following LPS stimulation between healthy controls and MIS-C patients (Fig. 1B). CD11b expression in monocytes was not statistically different when compared between controls and children with MIS-C at basal levels or after stimulation with LPS (Fig. 1C). Monocyte CD11b expression was not different between healthy controls and children with MIS-C at baseline or after LPS stimulation (Fig. 1C). Similar to neutrophils, TLR4 expression in the total monocyte population was not significantly different following LPS stimulation in children with MIS-C or when comparing controls to children with MIS-C (Fig. 1D).

**Figure 2:**
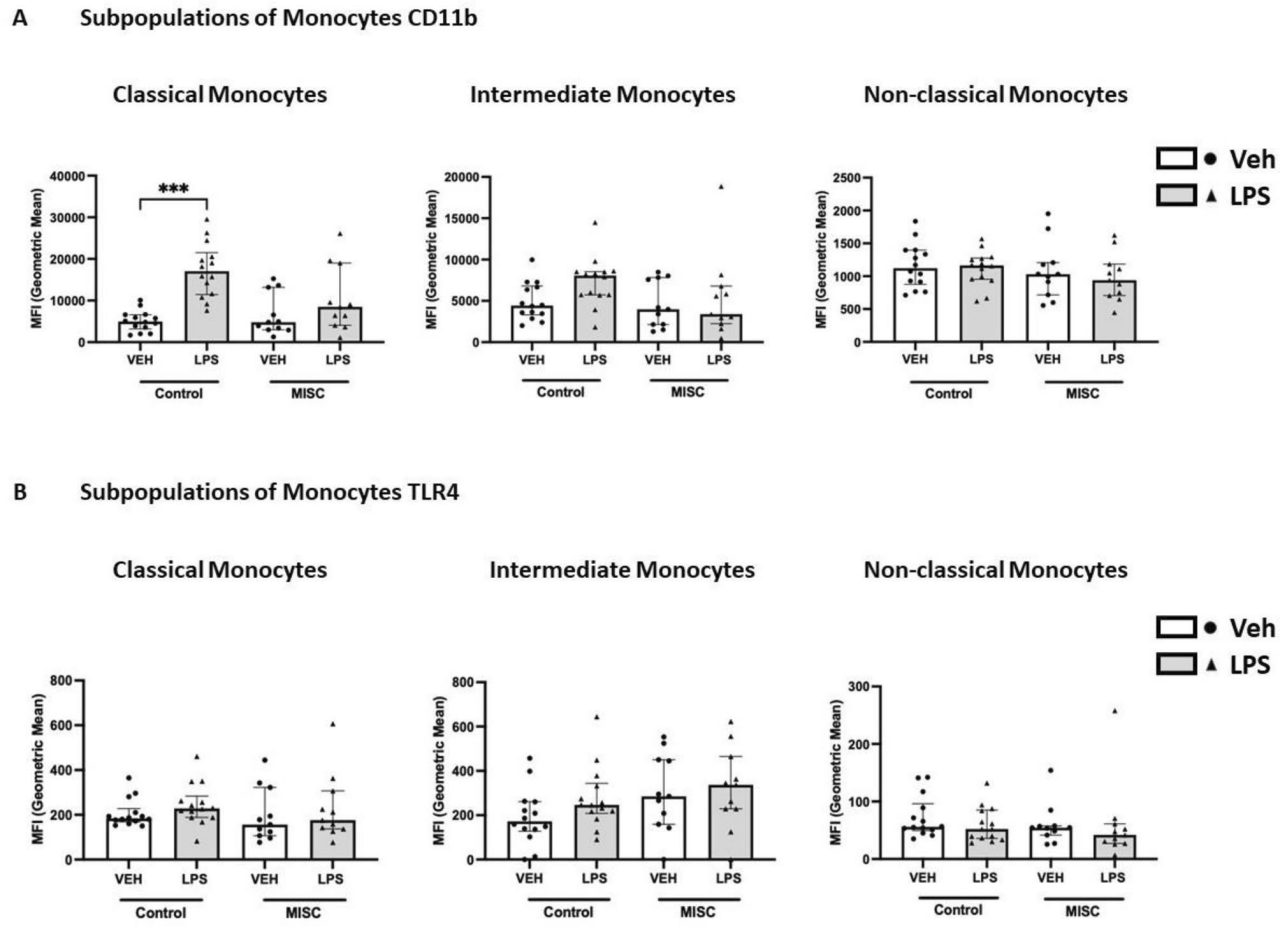
Expression of CD11b and TLR4 in subpopulations of monocytes in healthy controls and Multisystem inflammatory syndrome in children (MISC) patients: Whole blood from controls was stimulated with LPS (10 ng/mL) for 1 h at 37 °C, and monocyte markers were analysed by flow cytometry. Subpopulations were differentiated based on the expression of CD14 and CD16 into classical (CD14high CD16neg/low), intermediate (CD14high CD16high), and non-classical (CD14lowCD16high) (Fig. 1). (**A**) CD11b and (**B**) TLR4 expression in subpopulations of monocytes are shown as Geometric Mean Channel Fluorescence (MFI) with median and interquartile range (IQR); “veh” refers to unstimulated (PBS). Comparisons were done between two independent cohorts and both conditions (‘Veh’ and ‘LPS-treated’). ***p < 0.001, using Kruskal–Wallis test.

To further characterise the role of monocytes in the immune response of children with MIS-C, expression of CD11b and TLR4 were determined in the classical (CD14high CD16neg/low), intermediate (CD14high CD16high), and non-classical (CD14lowCD16high) subtypes of monocytes. CD11b expression significantly increased in classical monocytes following LPS stimulation in controls (*p* = 0.0001) but not in children with MIS-C (Fig. 2A). Expression of TLR4 in classical monocytes was not different when comparing LPS treatment in both groups. No differences in the expression of CD11b or TLR4 were found in classical, intermediate and non-classical monocytes following LPS treatment or when comparing children with MIS-C to controls (Fig. 2B). Children with MIS-C showed a tendency to LPS hyporesponsiveness for neutrophil and classical monocytes CD11b expression**.**

**Figure 3:**
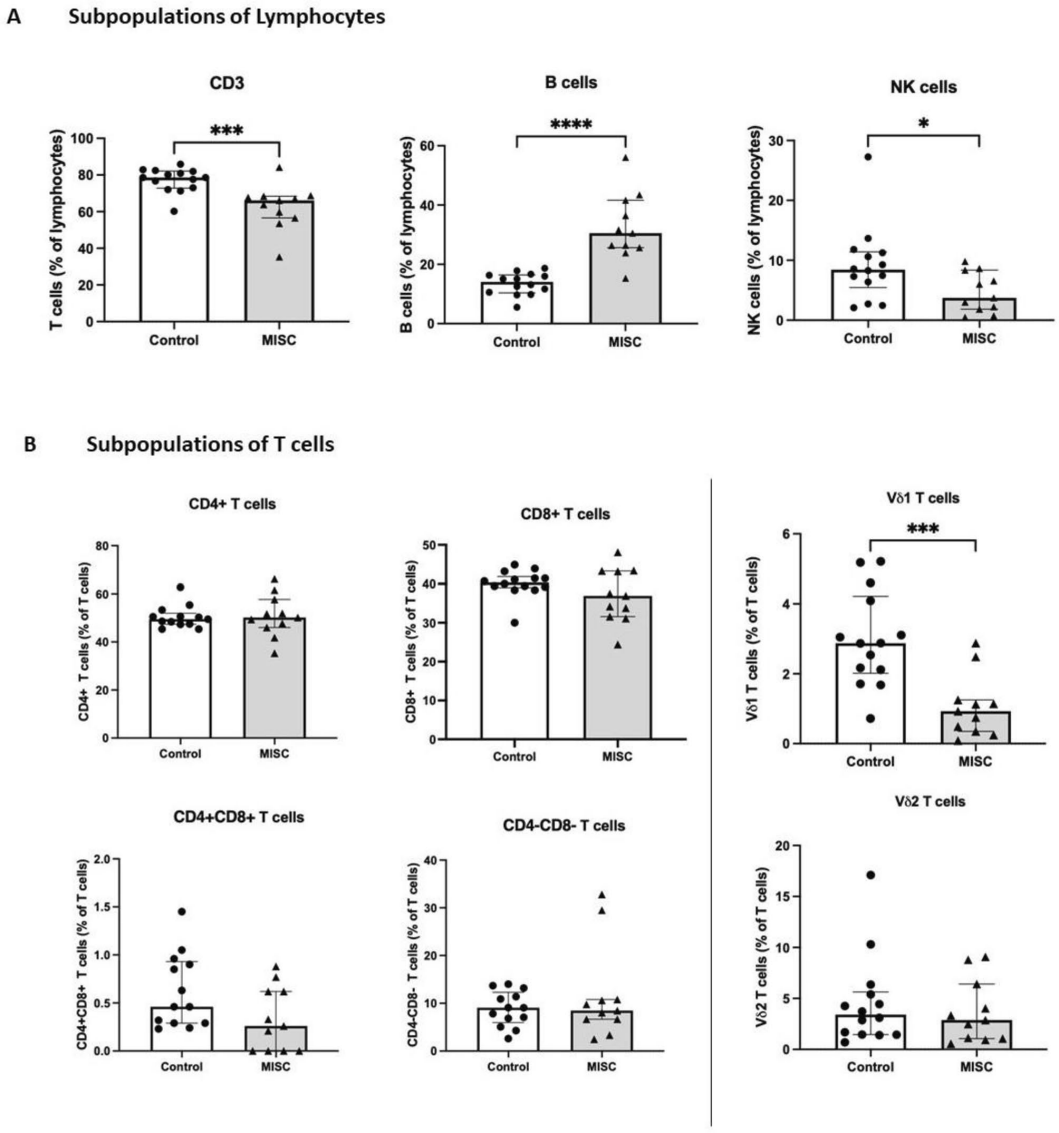
Frequencies of lymphocytes in healthy controls (n = 14) and Multisystem inflammatory syndrome in children (MISC) patients (n = 11). (**A**) Frequencies of T cell, B cell and NK cells. (**B**) Frequencies of CD4^+^, CD8^+^, CD4^+^CD8^+^ (double positive), CD4^−^CD8^−^ (double negative), innate T cells—Vδ1 T cells and Vδ2 T cells. Results are presented as percentages with median and interquartile range (IQR). Comparisons were made between two independent cohorts. *p < 0.05, ***p < 0.001, ****p < 0.0001 using Mann Whitney test.

Frequencies of lymphocytes were determined by staining whole blood with antibodies specific for CD3, CD19 and CD56. Total T, B, and NK cells were enumerated as percentages of total lymphocytes (Figure S2). Figure 3A shows lower frequencies of T and NK cells in children with MIS-C compared to healthy controls (*p* = 0.0008 and *p* = 0.0333 respectively). In contrast, B cell frequencies increased in children with MIS-C (*p* < 0.0001). These results show that T, B and NK cell frequencies in peripheral circulation reflected systemic alterations of the immune response in children with MIS-C.

Further analysis of T cells subpopulations based on the expression of CD4 and CD8 positivity, allowed the identification of helper (CD4^+^ CD8^-^), cytotoxic (CD8^+^ CD4^-^) and unconventional (CD4^+^ CD8^+^ and CD4^−^ CD8^−^) T cells in both groups. The frequency of these subpopulations was determined as percentages of total CD3^+^ cells (Figure S2). No difference in the proportions of CD4^+^, CD8^+^, CD4^+^ CD8^+^ or CD4^−^ CD8^−^ T cells was observed when comparing children with MIS-C with healthy controls (Fig. 3B, left). Gamma-delta (γδ) T cells subsets, Vδ1 and Vδ2, were also evaluated as percentages of total CD3^+^ cells (Fig. 3B, right). Vδ1 T cells were significantly reduced in MIS-C patients compared to healthy controls (*p* = 0.0005), while no difference in Vδ2 subsets was observed between the children with MIS-C and the healthy control group. There was no change in T cell subtypes of helper, cytotoxic and unconventional Vδ2 T cells in the circulating blood of children with MIS-C.

### NLRP3 inflammasome activation and serum cytokine levels in healthy controls and children with MIS-C

**Figure 4:**
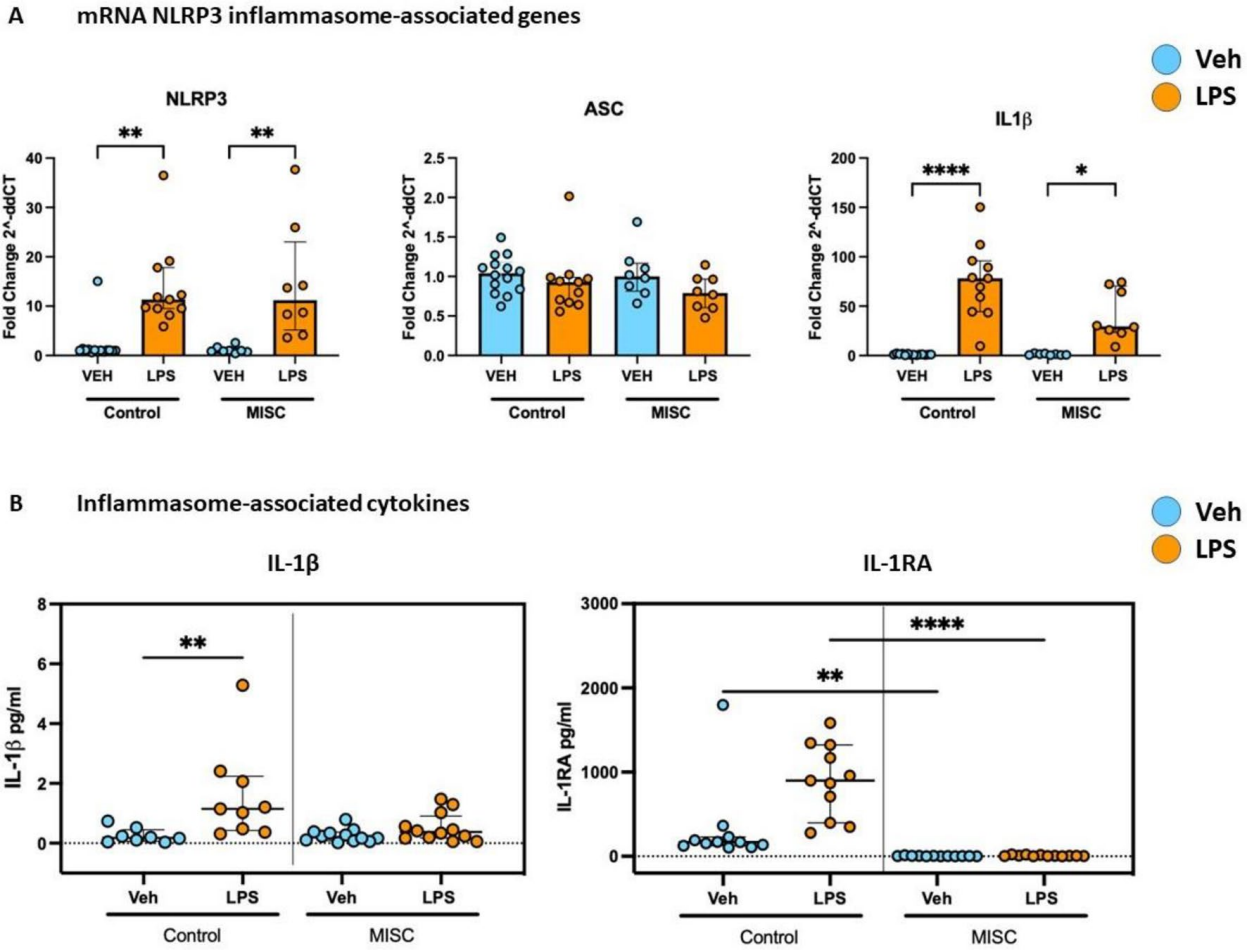
NLRP3 inflammasome activation was evaluated by analysing the (**A**) messenger RNA levels of NLPR3, ASC and IL-1β, and (**B**) protein expression of IL-1β and IL-1RA. Whole blood of healthy controls and Multisystem inflammatory syndrome in children (MISC) patients were stimulated with LPS (10 ng/mL) for 1 h at 37 °C. Samples were then processed for RNA analysis by PCR and cytokines expression in serum. (**A**) NLPR3, ASC and IL-1β were measured in total mRNA extracted from whole blood of healthy controls (Veh n = 14; LPS n = 11) and Multisystem inflammatory syndrome in children (MISC) patients (n = 8). (**B**) Cytokine concentrations of IL-1β (left) and IL-1RA (right) were measured by multiplex enzyme-linked immunosorbent assay (ELISA) in serum of healthy controls (n = 14) and Multisystem inflammatory syndrome in children (MISC) patients (n = 11). Results are presented as (**A**) fold change expression of NLRP3, ASC and IL-1β with median and interquartile range (IQR), and (**B**) pg/mL concentration with median and IQR; “veh” refers to unstimulated (PBS). Comparisons were done between two independent cohorts and both conditions (‘Veh’ and ‘LPS-treated’). *p < 0.05, **p < 0.01, ****p < 0.0001 using Kruskal–Wallis test.

Whole blood mRNA levels of two main components of the NLR family pyrin domain containing 3 (NLRP3) inflammasome, NLRP3 and Apoptosis-associated speck-like protein containing a CARD (ASC), and of one of its pro-inflammatory products, interleukin-1 β (IL-1β) were evaluated (Fig. 4A). NLRP3 mRNA levels increased after LPS stimulation in controls (*p* = 0.0011) and children with MIS-C (*p* = 0.0025) when compared to their corresponding untreated sample, but no difference was observed when groups were compared. No statistically significant difference in the levels of ASC mRNA was observed between untreated samples and LPS or between groups. mRNA expression of IL-1β was seen to increase following stimulation with LPS in both controls (*p* < 0.0001) and children with MIS-C (*p* = 0.0290).

As an indirect measurement of NLRP3 inflammasome activation, protein expression of the pro-inflammatory NLRP3 activated cytokine IL-1β, and anti-inflammatory IL-1 regulator cytokine, IL-1RA, were measured in serum samples from both groups by multiplex immunoassay (Fig. 4B). A significant increase in mature IL-1β expression following LPS stimulation was observed in controls (*p* = 0.0088) but not in children with MIS-C when compared to the corresponding untreated samples for each group (Fig. 4B, left). No significant difference was observed in IL-1β expression between children with MIS-C and controls when both treatments were evaluated. However, children with MIS-C seem to have impaired pro-inflammatory responses to LPS compared to healthy controls. In contrast, the anti-inflammatory response, measured by IL-1RA levels, was significantly reduced in children with MIS-C at both basal and LPS-treated samples when compared to controls (*p* = 0.0013 and *p* < 0.0001 respectively).

**Figure 5:**
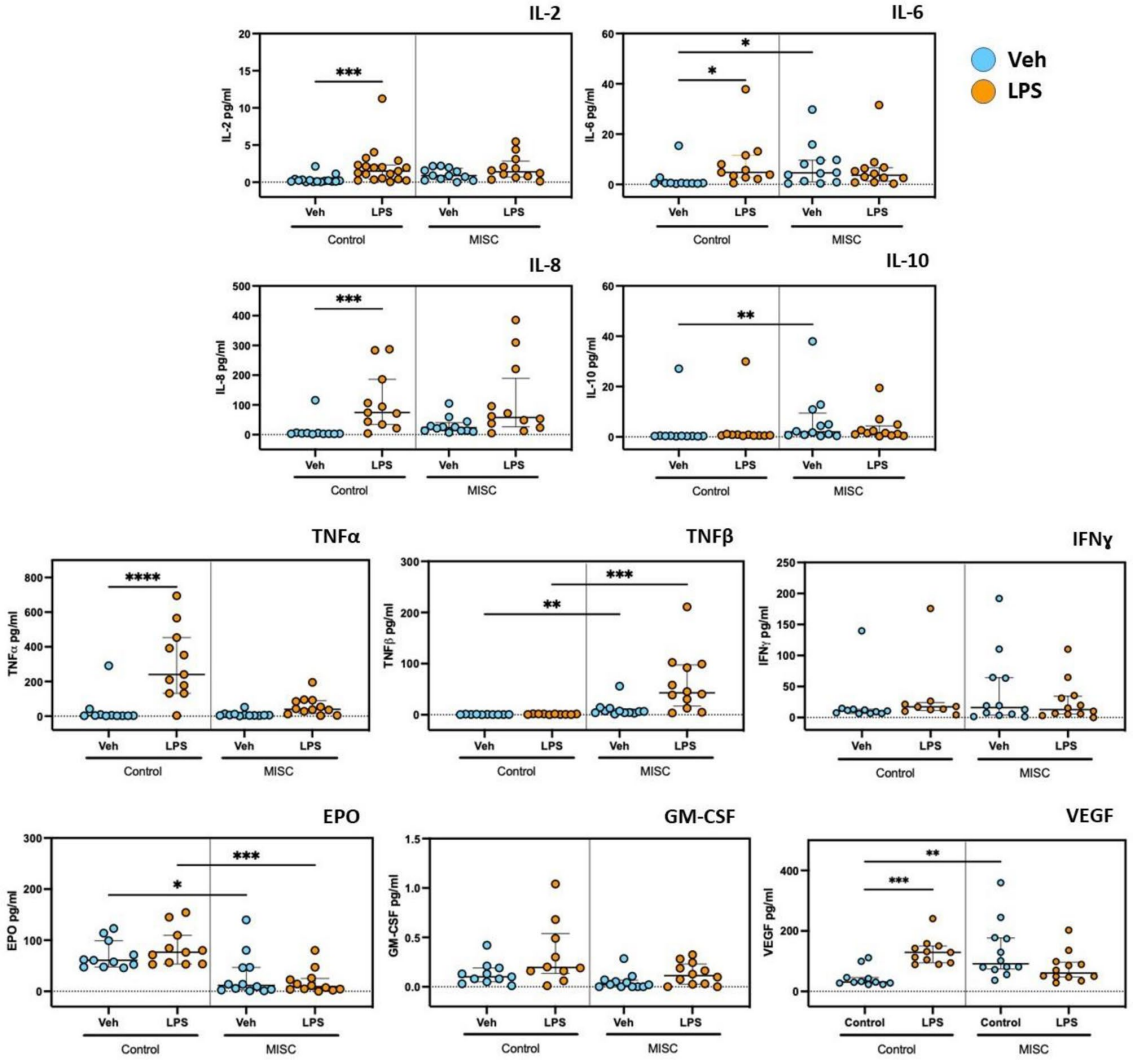
Cytokine response in healthy controls and Multisystem inflammatory syndrome in children (MISC) patients at baseline and on Lipopolysaccharides (LPS) stimulation: IL-2, IL-6, IL-8, IL-10, IFNɣ, TNFα, TNFβ, EPO, GM-CSF and VEGF. Whole blood was stimulated with LPS (10 ng/mL) for 1 h at 37 °C. Cytokine concentrations were measured by multiplex enzyme-linked immunosorbent assay (ELISA) in serum of healthy controls (IL-6, IL-8, IL-10, IFNɣ, TNFα, EPO, GM-CSF and VEGF n = 11; IL-2, n = 14; TNFβ, n = 10) and children with MISC (n = 12). Results are presented as pg/mL concentration with median and interquartile range (IQR); “veh” refers to unstimulated (PBS). Comparisons were done between two independent cohorts and both conditions (‘Veh’ and ‘LPS-treated’). *p < 0.05, **p < 0.01, ***p < 0.001, ****p < 0.0001 using Kruskal–Wallis test.

The levels of interleukin-2 (IL-2), IL-6, IL-8, IL-10, tumour necrosis factor-α (TNF-α), TNF-β, human interferon-γ (IFN-γ), granulocyte macrophage-colony stimulating factor (GM-CSF), vascular endothelial growth factor (VEGF) and erythropoietin (EPO) in serum samples controls were measured by multiplex immunoassay in both groups. Figure 5 shows the serum levels of these ten soluble molecules, which mediate the immune response, at basal level and after stimulation with LPS in the control group and children with MIS-C. Increased levels of IL-2 (*p* = 0.0009), IL-6 (*p* = 0.0153), IL-8 (*p* = 0.0004), TNF-α (*p* < 0.0001) and VEGF (*p* = 0.0003) were seen in healthy controls following LPS treatment. Increases in these cytokines were not observed in children with MIS-C after treatment with LPS.

Basal levels of IL-6 (*p* = 0.0468), IL-10 (*p* = 0.0029), TNF-β (*p* = 0.0020) and VEGF (*p* = 0.0056) were significantly higher in children with MIS-C when compared to basal levels in healthy controls. TNF-β levels (*p* = 0.0001) were also observed to be significantly increased in MIS-C patients compared to healthy controls after LPS treatment. In contrast, EPO was significantly reduced at basal level (*p* = 0.0406) and with LPS treatment (*p* = 0.0037) in children with MIS-C compared to controls. No differences were observed in IFN-γ and GM-CSF following LPS treatment or when comparing the groups.

## Discussion

Neutrophils are key players in both KD and COVID-19^18–21^. Neutrophil counts and neutrophil/lymphocyte ratio (NLR) are higher in children with MIS-C compared to children with COVID-19, and neutrophil counts have been suggested as a predictor of responsiveness to intravenous immunoglobulin therapy in patients with KD^4,7,9,13–16,19,20^. Our findings are in line with previous reports indicating high absolute counts of neutrophils and NLR, as well as low counts of absolute lymphocytes in children with MIS-C compared to normal ranges. Earlier studies have described significant increases of CD11b in MIS-C when compared to children with mild and severe COVID-19, but reduced expression of the marker when comparing children with COVID-19 and healthy controls^22^. Similarly, we observed a tendency to reduce CD11b in children with MIS-C after a secondary challenge with endotoxin, however, this did not reach significance, likely due to our sample size. Our results contrast those of *García-Salido *et al*.* (2022), who reported high baseline expression of CD11b in monocytes and neutrophils in children with MIS-C compared to healthy controls and in children with KD^23^. These differences could be due to the number of patients included in the studies and the different methodological approaches employed when defining neutrophil and monocyte populations by flow cytometry. Furthermore, contradictory results indicating decreased or increased expression of CD11b in neutrophils in KD compared to controls could suggest heterogeneity in the expression of this marker in the population and the need for a larger cohort of studies^23–25^.

Several studies have reported distinguishing features of uncontrolled neutrophil activation in children with MIS-C^5,6,26–30^. Through transcriptional profiling, *Boribong *et al*.* (2021) identified a clear signature of neutrophil activation, degranulation and spontaneous neutrophil extracellular trap (NET) formation^26^. Other activation markers such as Fc-γ receptor I (FcγRI; CD64) and CD54 (ICAM1) were upregulated in neutrophils, total monocytes and CD16 + non-classical monocytes in children with MIS-C when compared to controls^6,28,31^. Interestingly, we found CD11b expression in MIS-C did not increase after stimulation with LPS, as it did in controls. To our knowledge, no other study has described changes in neutrophil response in children with MIS-C following a secondary stimulation. However, evaluation of LPS levels in the plasma of children with MIS-C shows increased levels of this microbial marker compared to children with COVID-19 and controls^32^. Low-grade endotoxemia (presence of LPS) was also detectable in patients with SARS-CoV-2 infection and neutrophils of early onset KD patients^33–35^. Based on our results and these reports of LPS levels, it is difficult to conclude whether the response observed in our study is due to an altered secondary response by trained immunity or the presence of co-infection after SARS-CoV-2 infection. Further analyses are required to fully understand this interaction.

LPS receptor TLR-4 was proposed as one of the pathogenic mechanisms triggered by SARS-CoV-2 infection^36–38^. It has been suggested that TLR4 can directly bind the SARS-CoV-2 spike glycoprotein or can be activated by lung viral infection-derived subproducts such as oxidized phospholipids and alarmin S100A9^36–38^. We measured the cell surface expression of TLR4 in neutrophils and monocytes and found no statistical differences in the expression of TLR4 in any cell type, or when comparing the MIS-C patient group to the control group. To our knowledge, no other study has evaluated TLR4 in children with MIS-C to date. TLR4 and some of its mediated inflammatory signalling molecules are reported to be upregulated in severe COVID-19 patients^38,39^. *Root-Bernstein *et al*.* (2021) suggest TLR4's role in severe COVID-19 is due to synergistic immune activation induced by SARS-CoV-2-bacterium/fungus coinfection^39^. TLR4 may have a pathological role in COVID-19 inflammation, our data and other data published to date do not indicate a similar role in MIS-C.

Our data suggests an alteration in lymphocyte frequencies in children with MIS-C. Our total T cell and NK cell findings are in line with previous reports and could indicate migration to inflamed tissues^5,6,8,14,40,41^. *Gruber *et al*.* 2020 reported similar results when immunophenotyping peripheral blood of MIS-C patients and healthy controls by mass cytometry^6^. A transcriptome-wide analysis using RNA sequencing of peripheral blood of MIS-C patients found depletion of NK cells when compared to controls^42^. Increased frequencies of B cells have also been reported by others in acute MIS-C patients^14,29^. *Ramaswamy* et al. (2021) also reported more mutated IgG clones and lower B cell clonal diversity in these patients^29^. However, contrasting findings have been described, where total B cell counts were reduced with higher class-switched memory B cell counts in the MIS-C resolution phase compared with the acute phase^28^.

When analysing the frequencies of helper (CD4^+^ CD8^−^), cytotoxic (CD8^+^ CD4^−^) and unconventional CD4^+^ CD8^+^, CD4^−^ CD8^−^ and gamma-delta (γδ: Vδ1 and Vδ2) T cells subsets, we saw reduced Vδ1 T cells in the MIS-C patients when compared to controls. Reduced helper (CD4^+^), cytotoxic (CD8^+^) and γδ T subsets have been described in the acute phase of MIS-C^5,6,8,28,40^. *Carter *et al*.* (2020) reported high frequencies of CD4^+^CCR7^+^ T cells and lower γδ T cells in the acute phase of MIS-C patients as well as more regulatory T cell counts in the convalescence phase^28^. *Ramaswamy *et al*.* 2021 also described high proportions of regulatory T cells and decreased helper (CD4^+^) T cells in the peripheral blood of MIS-C patients compared to controls^29^. The expansion of the Vβ21.3 T cell receptor β chain in both CD4 and CD8 T cell subsets indicates an activated phenotype in children with MIS-C^43^. While memory CD8^+^ T are reported to be increased in patients, RNA sequencing analysis of blood from MIS-C patients indicates exhaustion of effector CD8^+^ T cells and potentially impaired cytotoxicity and pathogen-killing capacity^42^. Most of these changes in the adaptive immune response seem to normalize in the resolution and convalescence phases of MIS-C^28,43^.

NLRP3 inflammasome activation is a crucial mechanism of the innate immune system against invading pathogens. Canonical activation of NLRP3 requires priming by Toll-like receptors (TLR) ligands such as LPS, and assembly of a multiprotein complex (NLRP3, ASC and Caspase 1) which in turn mediates proteolytic processing of pro-interleukin-1β (IL-1β) and pro-IL-18, inducing inflammation. *Wang* et al. (2021) used whole blood microarray mRNA analysis to show a significant increase of NLRP3, Caspase 1, and IL-1β, as well as some noncanonical inflammasome markers (Caspase 4 and Caspase 5) in acute KD patients and MIS-C when compared to convalescent controls (healthy children with previous history of KD)^44^. They suggest a strong tendency towards caspase-4/5-dependent noncanonical inflammasome activation in MIS-C patients which could eventually trigger caspase-1-dependent NLRP3 inflammasome activation^44^. Our results show an increase in NLRP3 mRNA after LPS, but not in ASC mRNA, in both healthy children and children with MIS-C, but no difference between groups. Increased protein expression of cytokine IL-1β and IL-18 is an indirect indication of NLRP3 inflammasome activation, while anti-inflammatory IL-1 (IL-1RA) provides information about its regulation. In this study, an increase in mature IL-1β and IL-1RA was observed in healthy controls after LPS stimulation, as expected in a healthy pro-inflammatory response. This was not observed in children with MIS-C, which indicates an alteration in NLRP3 inflammasome activation in these patients. No statistical differences in the expression of mature IL-1β or IL-1β mRNA were observed in our cohort of MIS-C patients when compared to controls, however, a tendency towards reduction was seen. IL-1β protein expression in our study contrasts with reports from other research groups showing not only an increase in IL-1β but also of IL-18, and a strong association between IL-1β levels and a more severe disease course^27,28,43,45^. Furthermore, our data indicates the downregulation of anti-inflammatory IL-1RA in MIS-C children at both basal level and in LPS-treated samples compared to healthy controls. The opposite results have been previously described by other studies, where IL-1RA was increased in serum or plasma of children with MIS-C^14,43,45^.

Soluble molecules are important mediators of immune response and can trigger the activation or inactivation of specific cell types or molecular responses. We measured the levels of a panel of cytokines associated with pro- and anti-inflammatory signalling pathways. A significant increase in basal levels of IL-6, IL-10, TNF-β and VEGF in the serum of children with MIS-C compared to healthy controls was observed. These findings are in line with previous reports of an increase in IL-6 and IL-10 in MIS-C patients^6,14,27,28,40,43,45–48^. Our data contrasts with others in the expression of IL-8, TNF-α, and IFNγ. We found no difference while an increased level in MIS-C children was seen in these studies^27,28,43,45,47–49^. *Syrimi *et al*.* found no differences in the levels of these cytokines/chemokines when comparing patients to healthy controls^14^. To date, no other reports are showing an increase in TNF-β and VEGF. Many other soluble markers are consistently high in MIS-C patients when compared to healthy controls or COVID-19 patients, including cytokines IL-2, IL-2RA, IL-15, IL-and 17A, cytokine receptors sIL-2R, Tumor necrosis factor receptor 1 (sTNF-R1) and sTNF-R2, chemokines CCL2, CCL3, CCL4, CCL20, CCL28, CDCP1, CXCL9 and CXCL10, and soluble markers sCD40L, plasminogen activator inhibitor 1 (PAI-1), pentraxin-3 (PTX3), myeloperoxidase (MPO), monocyte chemoattractant protein (MCP)-1, MCP-2, macrophage-inflammatory protein (MIP)-1α and MIP-1β^6,14,40,43,45,46^. Discrepancies in cytokines expression profiles are likely due to the number of patients recruited, characteristics of the cohorts, methodological approaches, and the timing of sample collection. A clear profile of cytokines would allow clinicians to discriminate between patients with MIS-C and COVID-19 patients, or between different grades of severity in MIS-C patients^5,46,47^. *Caldarale *et al*.* (2021) suggest that the pattern of cytokines/chemokines in MIS-C leans toward a Th1-type immune response^48^. Altogether, the MIS-C soluble markers profile seems to indicate inflammation, favour lymphocytic and myeloid activation, and mucosal immune dysregulation^5,6,49^.

Interestingly, when we exposed whole blood of MIS-C patients to endotoxin challenge we saw no increase in levels of IL-2, IL-6, IL-8, TNF-α and VEGF as was observed in healthy controls. There is a marked hyporeactivity in MIS-C patients to LPS. Increased TNF-β and reduced EPO were the only significant variations in children with MIS-C following LPS treatment compared to controls. TNF-β levels have not been reported to be increased in a MIS-C cohort so far, especially with such a considerable difference after LPS treatment. However, other members of the TNF superfamily of molecules such as TNF-α and sCD40L, and receptors sTNF-R1 and sTNF-R2 were found to be increased at basal levels in a MIS-C^14,27,43,45,47^. In KD, TNF-α and TNF-β levels have been reported to be high intracellularly in monocytes and lymphocytes of patients in the acute stage, but TNF-β reduced in serum^50,51^. In contrast to TNF-β, EPO expression was low in children with MIS-C compared to controls for both basal levels and after LPS. So far, changes in EPO levels have not been described in this cohort by other studies. Considering its anti-inflammatory, anti-apoptotic and protective function, EPO has been suggested as a potential treatment for COVID-19^52,53^. However, no clear results of its therapeutic efficacy for COVID-19 were found. Further studies are required to fully understand the role of TNF-β and EPO in the pathogenesis of MIS-C.

This study identified a decreased activation of neutrophils and monocytes when evaluated by CD11b expression, and reduced levels of pro-inflammatory cytokines in response to endotoxin challenge in MIS-C. Further studies are required to fully understand the pathological immune responses in MIS-C and to identify potential immunomodulatory therapies.

## Methods

### Ethical approval and study participants

Ethical committee approval for the study of blood samples from children was granted by the National Research Ethics Committee for COVID-19-related Research (NREC COVID-19; 20-NREC-COV-032). All experiments were performed following relevant guidelines and regulations. Informed written consent was obtained from all parents of children who took part in the study.

Blood and serum samples were obtained from healthy school-age children (aged 5–15 years) undergoing phlebotomy as part of a day case procedure and children with MISC (aged 5–13 years) admitted to CHI. Healthy controls were having minor surgical procedures. Children with medical co-morbidities or chronic conditions were excluded from being healthy controls. Case definition for MIS-C was described as per recommendations of Centers for Disease Control and Prevention (CDC): individual aged < 21 years, presenting with fever (> 38.0 °C for ≥ 24 h), laboratory evidence of inflammation, clinically severe illness requiring hospitalization, multisystem (> 2) organ involvement (cardiac, renal, respiratory, hematologic, gastrointestinal, dermatologic or neurological), no alternative plausible diagnoses. A recent positive history of SARS-CoV-2 infection or exposure to a suspected or confirmed COVID-19 case 4 weeks before the onset of symptoms was included but not necessarily used as a diagnostic criterion.

### Blood sampling

Whole-blood sampling was performed following informed parental consent Sampling was performed using an aseptic technique via central and peripheral arterial lines and venous sampling at times of routine patient phlebotomy. The blood (1–3 mL) was collected in sodium citrate tubes and processed within 2 h of collection.

### Granulocytes cell surface antigen expression

100µL of whole blood was incubated at 37 °C for 1 h (5% CO_2_) in the presence or absence of Lipopolysaccharides (LPS) at a final concentration of 10 ng/ml (SIGMA Life Science, Ireland). Phosphate-buffered saline (PBS) was used in unstimulated samples (defined as “vehicle”) at an equal volume to LPS. Cells were then stained for 10 min at room temperature with monoclonal antibodies (mAb) specific for CD66b-Pacific Blue (6 µg/mL; G10F5; BioLegend, USA), CD14-PerCP (6 µg/mL; HCD14; BioLegend, USA), CD15-PECy7 (4 µg/mL; W6D3; BioLegend, USA), CD16-FITC (8 µg/mL; B73.1; BioLegend), USA, TLR4-APC (4 µg/mL; HTA125; BioLegend, USA), and CD11b-PE (1 µg/mL; D12; BD Biosciences, UK). After staining, cells were lysed in 1 mL FACS lysis buffer (BD Biosciences, Oxford, UK). Finally, the cells were washed with PBA buffer (PBS containing 0.1% fetal calf serum and 0.13% sodium azide), fixed with 1% paraformaldehyde (PFA) and analysed on a BD FACSCanto II flow cytometer and analysed with FlowJo software v10.8.1 (Tree Star, Ashland, USA). BD FACSCanto II performance check is done once a week using BD FACSDiva™ CS&T Research Beads (655,050; BD Biosciences, UK), and internal control of compensation is performed every time samples were acquired, using BD™ CompBeads Anti-Mouse Ig, κ/Negative Control Compensation Particles Set (552,843; BD Biosciences, UK). Samples were processed individually based on the time of collection. Neutrophils were defined as FSC-A and CD66b+ in single-cell gate and total granulocytes. Monocytes based on FSC-A, CD66b− , CD16+ and CD14+ . Monocytes were subdivided into classical (CD14high CD16neg/low), intermediate (CD14high CD16high), and non-classical (CD14lowCD16high) subtypes. CD11b and TLR4 were used as markers of activation. A minimum of 30,000 events were collected, and the relative expression of antigens was expressed as Geometric Mean Channel Fluorescence (MFI). The gating strategy used is shown in Figure S1.

### Lymphocyte subset phenotyping

100µL of whole blood were stained for 10 min at room temperature with monoclonal antibodies specific for CD3-Pacific Blue (4 µg/mL; HIT3a; BioLegend, USA), CD4-PE-Cy7 (4 µg/mL; SK3/Leu3a; BD, USA), CD8-PerCP (4 µg/mL; SK1; BioLegend, USA), CD19-APC (1 µg/mL; SJ25C1; BioLegend, USA), CD56-APC-Cy7 (2 µg/mL; 5.1H11; BioLegend, USA), Vδ1-FITC (3 µg/mL; TS8.2; Invitrogen, USA) and Vδ2-PE (1 µg/mL; B6; BioLegend, USA) T cell receptors (TCR). MAbs were diluted to pre-determined concentrations in PBA buffer (PBS containing 0.1% fetal calf serum and 0.13% sodium azide). After staining, cells were lysed in 1 mL FACS lysis buffer (BD Biosciences, Oxford, UK). Finally, the cells were washed with PBA buffer (PBS containing 0.1% fetal calf serum and 0.13% sodium azide), fixed with 1% paraformaldehyde (PFA) and analysed on a BD FACSCanto II flow cytometer and analysed with FlowJo software v10.8.1 (Tree Star, Ashland, USA). BD FACSCanto II performance check is done once a week using BD FACSDiva™ CS&T Research Beads (655,050; BD Biosciences, UK), and internal control of compensation is performed every time samples are acquired, using BD™ CompBeads Anti-Mouse Ig, κ/Negative Control Compensation Particles Set (552,843; BD Biosciences, UK). Samples were processed individually based on the time of collection. T, B and NK cell percentages were presented as their corresponding proportions in Lymphocytes only (100% of lymphocytes calculated based on the number of T cells+ , B cells+ and NK cells). NK cells were defined as CD3^−^ CD56^+^ cells, and B cells were defined as CD3^-^ CD19^+^ cells. T cells were defined as CD3^+^ cells. Within the CD3^+^, fours subpopulations were defined based on the expression of CD4 and CD8: CD4 T cells (CD4^+^ CD8^−^), CD8 T cells (CD4^−^ CD8^+^), Double positive T cells (CD4^+^ CD8^+^) and Double negative T cells (CD4^−^ CD8^−^). Vδ1 and Vδ2 T cells were defined as cells expressing CD3 and the Vδ1 or Vδ2 TCRs, respectively. The gating strategy used for enumeration of the different cell populations is shown in Figure S2.

### Quantitative real-time polymerase chain reaction (qRT–PCR) and analysis

300µL of whole blood was incubated at 37 °C for 1 h in the presence or absence of LPS at a final concentration of 10 ng/ml. Phosphate-buffered saline (PBS) was used in unstimulated samples (defined as “vehicle”) at an equal volume to LPS. After incubation, 1.3 mL of RNA*later*™ Solutions (Thermo Fisher Scientific, Waltham, MA, USA) was added to the whole blood, mixed, and then stored in a – 80 °C freezer until analysis. Samples were processed at the same time for RNA extraction, amplification and RT-PR analysis. RNA was extracted using a Ribopure blood kit (Thermo Fisher Scientific, Waltham, MA, USA), following the manufacturer’s instructions. RNA purity and concentration were determined using the NanoDrop ND-100 spectrophotometer and analysed using ND-1000 version 3.1.2 software. qPCR was performed using TaqMan primer probes (NLRP3, ASC and IL-1β); the evaluation of gene expression was performed by TaqMan® RT–PCR. Glyceraldehyde 3-phosphate dehydrogenase (GAPDH) was used as an endogenous control for data normalization. The 7900HT fast RT–PCR system (Thermo Fisher Scientific) was used, and relative quantification (RQ) values were calculated using the 2 − ΔΔCt method^54^. Fold change was calculated using the baseline control cohort average ΔCT as the calibrator for this calculation.

### Measurement of serum cytokine levels

Whole blood was incubated in the presence or absence of lipopolysaccharide (LPS) at a final concentration of 10 ng/ml (Sigma Life Science, Dublin, Ireland) at 37 °C for 1 h. The volume of blood used was variable, depending on the volume available per person, at least 200 µL used. Basel levels are defined as the concentration of cytokines without stimulation. Phosphate-buffered saline (PBS) was used in unstimulated samples (defined as “vehicle”) at an equal volume to LPS. After incubation, the serum was separated by centrifugation and stored at − 80 °C for later batch processing. Serum cytokine levels were analysed using a customized multiplex cytokine array by Meso Scale Discovery (Manchester, UK) and analysed on the Sector imager and validated (Meso Scale Discovery). The multiplex assay included mature interleukin-1 beta (IL-1β), interleukin-2 (IL-2), interleukin-6 (IL-6), interleukin-8 (IL-8), interleukin-10 (IL-10), interleukin-I receptor antagonist (IL-Ira), tumour necrosis factor-alpha (TNF-α), tumour necrosis factor -beta (TNF- β), interferon-gamma (IFN-Υ), vascular endothelial growth factor (VEGF), erythropoietin (EPO) and granulocyte–macrophage/colony-stimulating factor (GMCSF). This method employs a 96-well sandwich immunoassay which can quantify up to 12 analytes in 25 µl samples. The plate was then analysed on the SECTOR Imager and validated (Meso Scale Discovery, Rockville, MD, USA; www.mesoscale.com). Sensitivities were < 1 pg/ml for many assays. All assays used the same diluents, diluting linearly from one to fourfold. Non-specific binding between assays was typically < 0.1%. The assay reproducibility was calculated using the intra-variation of the standard curves and was shown to be within an acceptable range. The lower limit of detection for all analytes were as follows: EPO, GM-CSF, IFN-γ, IL-10, IL-18, IL-1RA, IL-1α, IL-1β, IL-2, IL-6, IL-8, TNF-α, TNF-β, VEGF, 0.89, 0.11, 5.17, 0.17, 1.15, 1.93, 0.39, 0.07, 0.71, 0.22, 0.11, 0.55, 0.23, 8.7 pg/ml respectively. Where the sample was below the lower limit of detection the value of the lower limit of detection for that assay was substituted^55^. All reported results units are in pg/ml.

### Statistical methods

Statistical analysis was performed on GraphPad PRISM version 9 using unpaired (unmatched), non-parametric Kruskal–Wallis and Mann Whitney test to compare results between two independent cohorts and both conditions, ‘Veh’ and ‘LPS-treated’. The Kolmogorov–Smirnov test was used to verify normality. Significance was defined as *p* < 0.05. Results shown are expressed as median and interquartile range (IQR). *p < 0.05, **p < 0.01, ***p < 0.001, ****p < 0.0001. *p-value* is not present means Not significant (ns) *p* > 0.05.

### Supplementary Information

Supplementary Information.

## Data Availability

Raw data was generated at The Children´s Research Laboratory, Discipline of Paediatric Medicine, Trinity College Dublin, the University of Dublin. Derived data supporting the findings of this study are available from the corresponding author (EJM) on request.
