# Supplementary material for: Innate immune dysregulation in multisystem inflammatory syndrome in children (MIS-C)
Source: Sci Rep. 2023 Sep 30;13:16463. doi: 10.1038/s41598-023-43390-6 (PMC10542373; doi:10.1038/s41598-023-43390-6)
Supplement: Supplementary file 1 — Supplementary Information. [file 41598_2023_43390_MOESM1_ESM.docx]

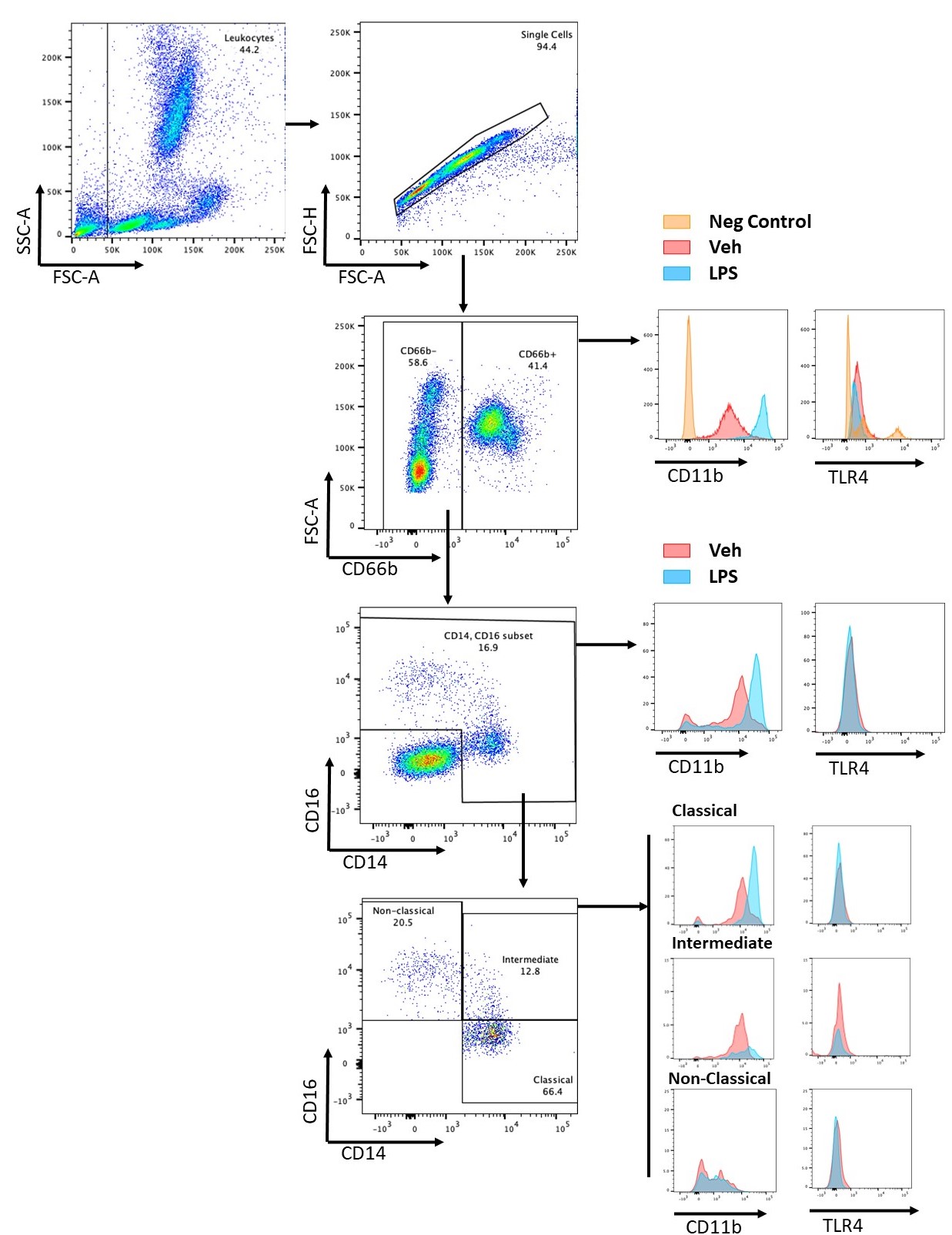


**Figure S1. Gating strategy for CD11b and TLR4 expression in neutrophils (CD66b+) and monocytes (CD66- CD14CD16) using flow cytometry**. Clockwise from the top left: Dot plot showing forward scatter area (FSC-A) plotted against side scatter area (SSC-A) with a gate drawn around the leukocytes; Dot plot showing FSC-A plotted against FSC-height (FSC-H) for gated leukocytes with a gate drawn around the single cells. Below single cells Dot plot, Dot plot showing FSC-A plotted against CD66b to select neutrophils (CD66b+); to the right, histograms showing expression of CD11b and TLR4 in CD66+ cells, overlays show Negative internal control (Lymphocytes), “Veh” and LPS-treated. Below FSC-A plotted against CD66b, Dot plot showing the expression of CD14 and CD16 in CD66b- cells, named as total monocytes; to the right, histograms showing expression of CD11b and TLR4 in total monocytes, overlays show “Veh” and LPS-treated. Below CD14 plotted against CD16, Dot plot differentiating Classical (CD14+CD16-), Intermediate (CD14+CD16+) and Non-Classical monocytes (CD14-CD16+) in Total monocytes only; to the right, histograms showing expression of CD11b and TLR4 in subpopulations of monocytes, overlays show “Veh” and LPS-treated.


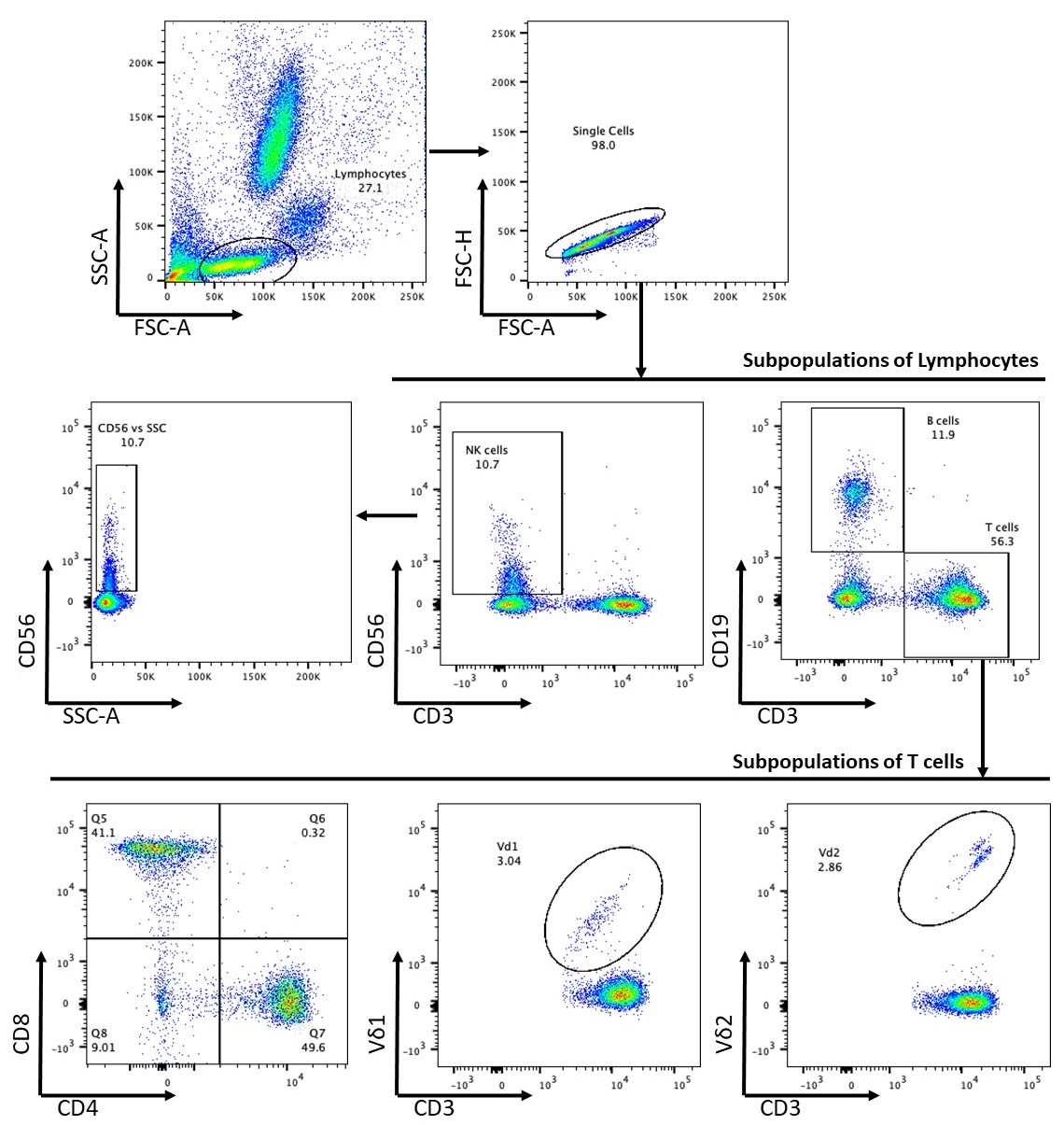


**Figure S2. Gating strategy for the detection of T cells, B cells, NK cells, CD4+ T cells, CD8+ T cells, CD4-CD8- T cells, CD4+CD8+ T cells, Vδ1 T cells and Vδ2 T cells by flow cytometry.** Whole blood was stained with monoclonal antibodies specific for CD3, CD4, CD8, CD19, CD56, Vδ1 and Vδ2 T cell receptors. Upper panels, left to right: flow cytometry dot plots showing forward scatter area (FSC-A) plotted against side scatter area (SSC-A) with a gate drawn around the lymphocytes; Dot plot showing FSC-A plotted against FSC-height (FSC-H) for gated lymphocytes with a gate drawn around the single cells. Centre panels, right to left: Three dot plots showing expression of CD3 plotted against CD19 (T and B cells), CD3 plotted against CD56 (NK cells), and SSC-A plotted against CD56 (NK cells control). Lower panels left to right: Dot plot showing expression of CD4 and CD8 by gated T cells with gates drawn around the CD4+ T cells, CD8+ T cells, double negative CD4-CD8- T cells and double positive CD4+CD8+ T cells; Dot plot showing expression of Vδ1 or Vδ2, gated T cells with a gate drawn around Vδ1 or Vδ2 cells.

|  | **WBC (x10^9/L)** | **Neutrophils (x10^9/L)** | **Lymphocytes (x10^9/L)** | **NLR** |
| --- | --- | --- | --- | --- |
| **1** | 11.3 | 7.4 | 2.4 | 3.0 |
| **2** | 12.2 | 10.2 | 1.8 | 5.7 |
| **3** | 5.8 | 5.0 | 0.7 | 7.6 |
| **4** | 4.2 | 3.2 | 0.5 | 6.2 |
| **5** | 11.1 | 9.8 | 0.5 | 20.8 |
| **6** | 11.9 | 11.0 | 0.6 | 20.1 |
| **7** | 7.7 | 6.8 | 0.3 | 25.3 |
| **8** | 16.5 | 13.3 | 1.9 | 7.2 |
| **9** | 11.0 | 8.5 | 1.3 | 6.4 |
| **10** | 19.2 | 14.7 | 2.2 | 6.6 |
| **11** | 5.7 | 4.5 | 0.4 | 12.4 |
| **12** | 14.8 | 8.4 | 4.1 | 2.1 |
| **13** | 9.0 | 7.1 | 1.6 | 4.5 |
| **14** | 10.4 | 8.2 | 1.2 | 6.8 |
| **15** | 4.1 | 3.1 | 0.4 | 7.8 |
| **16** | 21.6 | 19.0 | 1.0 | 19.8 |
| **17** | 11.2 | 9.3 | 0.8 | 12.4 |
| **18** | 24.7 | 20.3 | 3.2 | 6.3 |
| **AVER** | 11.8 | 9.4 | 1.4 | 10.0 |
| **STDEV** | 5.8 | 4.8 | 1.1 | 6.9 |
| **Aggregate (%)** | 33.3 | 61.1 | 61.1 | 94.4 |

**Table S1. Neutrophil to Lymphocyte ratio analysis.** Abbreviations and normal reference values: White Blood Cell (WBC) 5.0 – 12.0 x10^9^/L, Neutrophils 1.5 – 8.0 x10^9^/L, Lymphocytes 1.5 – 9.5 x10^9^/L, Neutrophil-to-lymphocyte ratio (NLR) > 3 abnormal/high. **Reference values from RCPCH REFERENCE RANGES- 2016.
